# Distributed Acoustic Sensor Using a Double Sagnac Interferometer Based on Wavelength Division Multiplexing

**DOI:** 10.3390/s22072772

**Published:** 2022-04-04

**Authors:** Andrey A. Zhirnov, Tatyana V. Choban, Konstantin V. Stepanov, Kirill I. Koshelev, Anton O. Chernutsky, Alexey B. Pnev, Valeriy E. Karasik

**Affiliations:** 1 Bauman Moscow State Technical University, 2-nd Baumanskaya 5-1, 105005 Moscow, Russia; a.zh@bmstu.ru (A.A.Z.); chobantv@yandex.ru (T.V.C.); koshelev-k@yandex.ru (K.I.K.); chernutsky.a@bmstu.ru (A.O.C.); pniov@bmstu.ru (A.B.P.); karassik@bmstu.ru (V.E.K.); 2 Kotelnikov Institute of Radioengineering and Electronics of RAS, Mokhovaya 11-7, 125009 Moscow, Russia

**Keywords:** Sagnac interferometer, fiber optic sensor, disturbance localization, sensitivity

## Abstract

We demonstrated a fiber optic distributed acoustic sensor based on a double Sagnac interferometer, using two wavelengths separated by CWDM modules. A mathematical model of signal formation principle, based on a shift in two signals analysis, was described and substantiated mathematically. The dependence of the sensor sensitivity on a disturbance coordinate and frequency was found and simulated, and helped determine a low sensitivity zone length and provided sensor scheme optimization. A data processing algorithm without filtering, appropriate even in case of a high system noise level, was described. An experimental study of the distributed fiber optic sensor based on a Sagnac interferometer with two wavelengths divided countering loops was carried out. An accuracy of 24 m was achieved for 25.4 km SMF sensing fiber without phase unwrapping.

## 1. Introduction

Distributed fiber optic sensors (FOS) have become quite popular for pipeline monitoring and perimeter security, etc., due to their ability to interrogate a long sensor length at a high spatial resolution and sampling rate. For recording high-frequency vibration influences, the most popular scheme is a phase-sensitive reflectometer, which provides a high accuracy of disturbance localization but has disadvantages such as a complex scheme and a high cost of components. A distributed sensor based on a Sagnac interferometer (SI) is gaining popularity due to its simple optical scheme, signal processing, and low dependency on laser phase noise [1]. In contrast with reflectometers, the possibility of using distributed measurements in schemes involving SI is not a default method. Such sensors are currently developing rapidly and there are three main ways to implement distributed sensing. The first is the “null frequencies” method, based on the position of the minima in the spectrum of the recorded signal from one loop of the interferometer [2]. The second is the expected time delay method, based on the correlation of arrays generated from two signals from loops of different known lengths or with another variant of delay [1]. The third method uses the time delay method between oppositely directed sensory loops [3]. However, the literature does not cover the issue of studying the sensitivity features of such systems. Moreover, creating a system with a sensor length of more than 20 km, which demonstrates a disturbance localization error of less than 0.1% of a total sensing length, especially in the case of a high noise level, requires using expensive components, and applying phase unwrapping and filtering. This makes data processing more complex and increases the cost of a sensor. Therefore, the issue of implementing a system with a simple optical scheme and low-cost components, which does not employ a complex data processing algorithm, and can operate at a high noise level, remains relevant.

**Figure 1:**
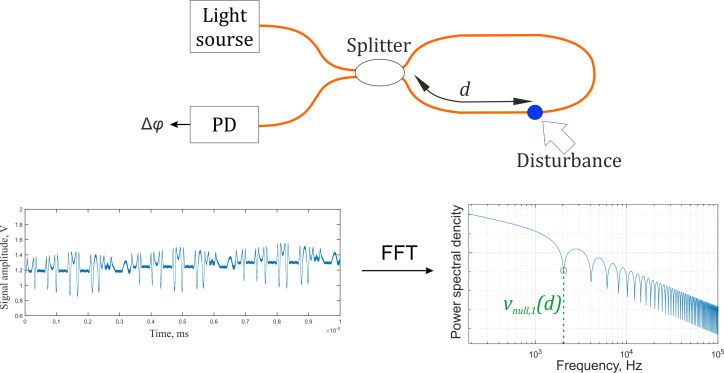
Diagram of a “null frequency” method principle.

The “null frequency” method is based on signal spectrum analysis from the sensor and the determination of a disturbance location using the low spectral density points’ positions. They appear in a spectrum in the presence of an acoustic disturbance due to a time delay between clockwise (CW) and counterclockwise (CWW) radiation. This method can be implemented based on one fiber loop [2], demonstrated in Figure 1, or in more complex schemes [4,5] such as the double Sagnac scheme [6], or another scheme in which a linear section and a Faraday rotator mirror (FRM) form at the output of the imbalanced Mach-Zehnder interferometer (MZI), with a laser coherence length less than the MZI arms’ difference [7,8,9,10]. In addition, the “null frequency” method was mentioned as an example for comparison in [1,5]. In [8], to reduce localization errors, the authors proposed a modified “null frequency” method, which consisted of applying the second Fourier transform to the signal spectrum in order to determine a periodicity of “null frequency” points instead of searching for individual ones. The error presented by the authors is 100 m for a sensor length of 50 km. A great disadvantage of these methods is that localization error strongly depends on a system noise level. Even after filtration, these methods are not appropriate for practical use, since the obtained spectra are distorted and unreliable. It is extremely difficult to determine the position of the low spectral density points or to find out the periodicity of spectrum modulation in the case of high noise. To provide good localization it is necessary to have some prior information about the position of the “null frequency” points, which is possible in a laboratory setup with a stated disturbance position assembled [3] or if the disturbance position can be obtained as a result of a numerical simulation [5].

**Figure 2:**
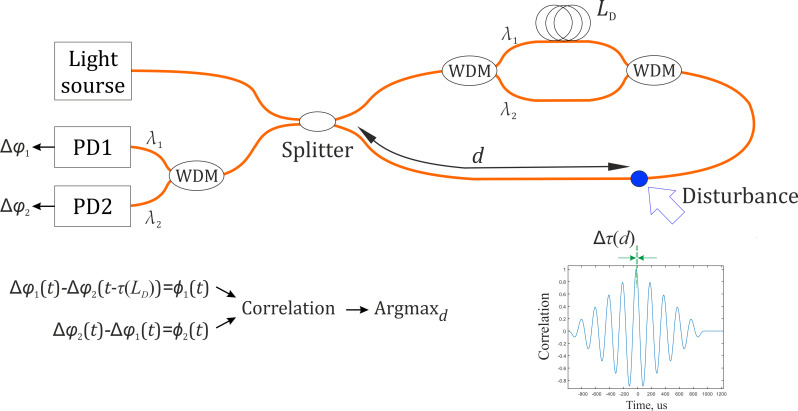
Diagram of a time delay estimation method principle.

The second method of implementing a distributed acoustic FOS based on a Sagnac interferometer is a time delay estimation. This requires a scheme with two loops that differ from each other by an fixed length amount *L_D_*. The scheme includes a MZI at one end of the loop [11,12,13], as shown in Figure 2, or a linear section with a mirror at the opposite edge [14,15], formed using couplers or WDM modules. The formed MZIs have a length difference *L_D_* between the arms that exceeds the coherence length of a laser source. Therefore, they do not create an interference signal and are only applied to separate optical paths and form two loops of different lengths. When using WDM modules, instead of splitters, radiation with a wavelength of *λ_1_* and *λ_2_* will propagate through the first and second loops [16,17]. Signals from the two loops are shifted relative to each other by a time delay, which is determined by the length *L_D_*. Since the loops have different lengths, each of the CW and CCW beams receives a phase shift from disturbance at different times. A demodulation data processing algorithm includes phase unwrapping of the signals from the two loops followed by correlation. Therefore, it is possible to determine the beginning moments of a disturbance in interference signals in each loop, calculate a time delay, and use it to find the location of the disturbance. A method of time delay introduction, implemented based on a scheme with one unbalanced MZI, allows the creation of a sensor with a length of 180 m [12] to 120 km [11]. In different studies, a disturbance position error was approximately a few hundreds of meters for a sensor length of 10 km (1% of the sensor length) [13], and up to 60 m for a sensor length of 120 km (0.05% of the sensor length) [11]. However, the method of time delay introduction has disadvantages. One disadvantage is a high noise level in a MZI due to an environmental influence, such as temperature fluctuations and vibrations. In addition, since in most cases a disturbance in the loop is quite intense, it becomes necessary to unwrap a signal phase. It requires at least two photodetectors for each loop, which increases the cost of the sensor and makes a data processing algorithm more complex.

**Figure 3:**
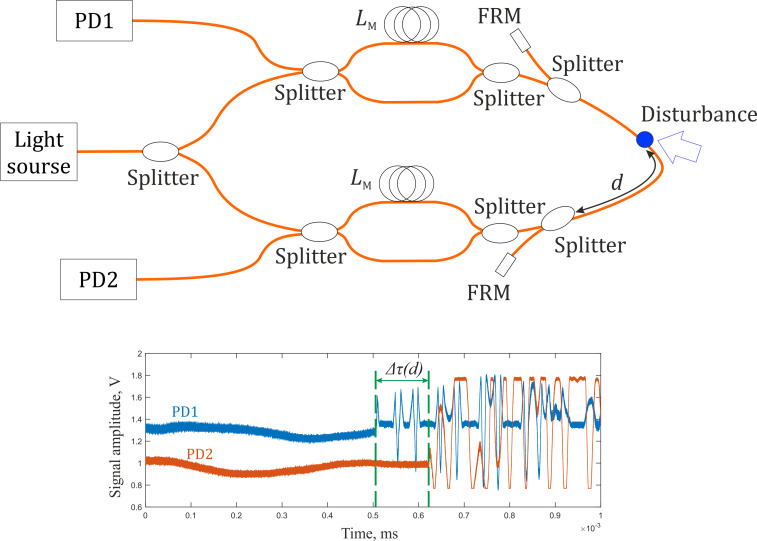
Diagram of a counter loops method principle.

One more method for disturbance localization is based on determining a time delay between signals from two counter loops of the same lengths. Two loops of a SI are formed, using MZIs with a difference *L_M_* between the arms that exceeds the coherence length of a radiation source, and Faraday mirrors (FRM) [3,18], as shown in Figure 3, or by using light polarization state multiplexing [19,20]. Thus, two SI loops are formed based on linear sections, which are located oppositely. As a result, in the presence of an acoustic disturbance on the common sensory fiber along which the radiation of both interferometers passes, a change in the interference signal is detected at time moments which differ from each other, which is the time delay. The value of the time shift between the signals is calculated by a correlation algorithm. However, the scheme with light polarization state multiplexing has significant disadvantages, as it includes expensive optical components, such as polarization-maintaining fibers, which contribute to the overall high cost and make it impractical for use on objects with a long perimeter. The scheme with a MZI and SI combination based on FRM has a great disadvantage of high loss. It has 4 fiber splitters that lead to a dynamic range of 18 dB (*Att* = 3 dB · 3 (couplers) · 2 (passes)), which can affect nonlinearity in propagating radiation and decrease signal power.

In this article, we proposed a distributed fiber sensor scheme based on a double Sagnac interferometer. The directions were separated by CWDM splitters that formed opposite loops of the Sagnac interferometer with equal lengths. These loops included two common fibers, one of which was a sensor, and the other a reference. The proposed scheme was promising in avoiding the disadvantages described above, as it was simple and did not need to use expensive fibers or a narrow-band laser. It demonstrated high disturbance localization accuracy even in the case of high noise level, and without phase unwrapping.

## 2. Theory of a Disturbance Localization Method

**Figure 4:**
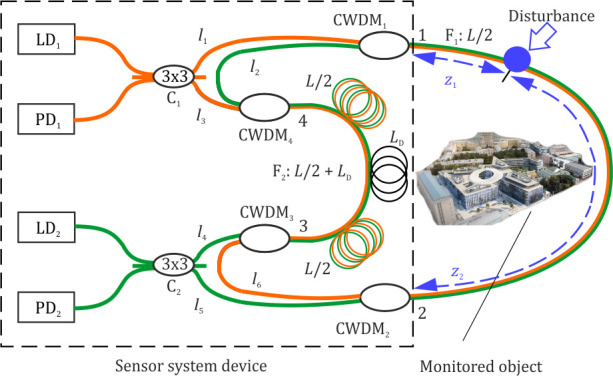
Structural scheme of Sagnac-based sensor system with two counter loops.

**Figure 5:**
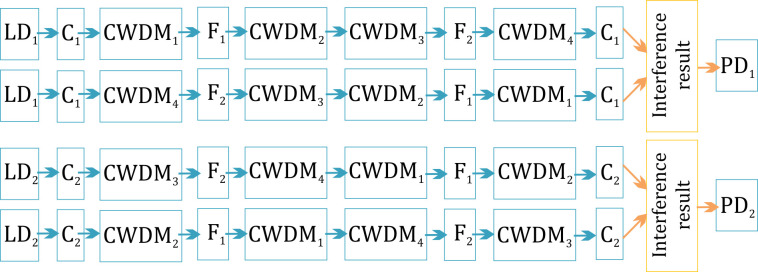
Possible light propagation paths.

The scheme of the proposed setup is shown in Figure 4. The principle of the sensor’s operation is as follows. Radiation from a continuous light source (LD_1),_ with wavelength (*λ*_1)_ in a range of 1540–1560 nm, enters the x-shaped fiber splitter (C_1)_, and is divided into two equal parts. We used a 3 × 3 splitter to avoid the Sagnac mirror effect that occurs when using a 2 × 2 splitter. Moreover, the 3 × 3 splitter provided a 2π/3 phase shift, which might be convenient for phase unwrapping in the scheme extension. One part of the light remaining after passing through splitter C_1_ propagated CW, and passed through CWDM_1_, the sensor fiber F_1_ in the 1→2 direction, CWDM_2_, CWDM_3_, the second reference fiber F_2_ in the 3→4 direction, and CWDM_4_. It then returned to the C_1_ splitter. The second part of the radiation, with a wavelength range of Δ*λ*_1_ = 1540–1560 nm and central wavelength *λ*_1_ = 1550 nm, passed through the interferometer loop CCW. It then passed through CWDM_4_, the F_2_ reference fiber in the 4→3 direction, CWDM_3_, CWDM_2_, the sensor fiber F_1_ in the 2→1 direction, and CWDM_1_, then passed through splitter C_1_, where interference occurred between the two parts of radiation with a wavelength *λ*_1_. After that, the interference result entered photodetector PD_1_. Similarly, in the second loop, radiation from a continuous light source (LD_2),_ with a wavelength range Δ*λ*_2_ = 1560–1580 nm and central wavelength *λ*_2_ = 1570 nm, entered the splitter C_2_ and was divided into two equal parts. One of the parts propagated CW through CWDM_3_, the reference fiber F_2_ in the 3→4 direction, CWDM_4_, CWDM_1_, sensor fiber F_1_ in the 1→2 direction, and CWDM_2_. The second part passed CCW through CWDM_2_, the F_1_ sensor fiber in the 2→1 direction, CWDM_1_, CWDM_4_, the F_2_ fiber in the 4→3 direction, and CWDM_3_. Then, both parts arrived at the C_2_ splitter. Following this process, photodetector PD_2_ detected the interference result. Due to signal forming features in a Sagnac interferometer, a point of low sensitivity arose at the half-length of each loop [21]. To move the points of low sensitivity outside the sensory region, we included an additional length *L*_d_ in fiber F_2_. The lengths of fibers *l*_1_ − *l*_6_ were negligibly small in comparison with *L* and *L*_d_ and were neglected in further calculations. Possible light propagation paths are shown in Figure 5.

**Figure 6:**
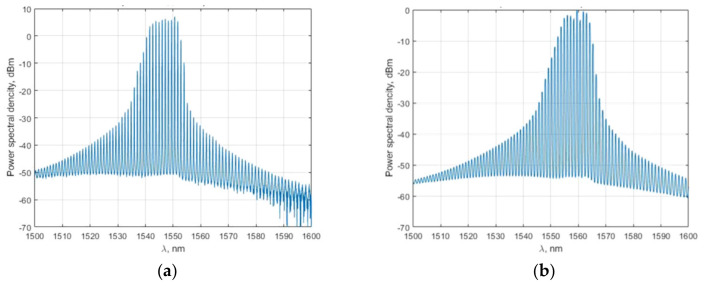
Spectra of Fabry-Perot laser diodes used in a system.

**Figure 7:**
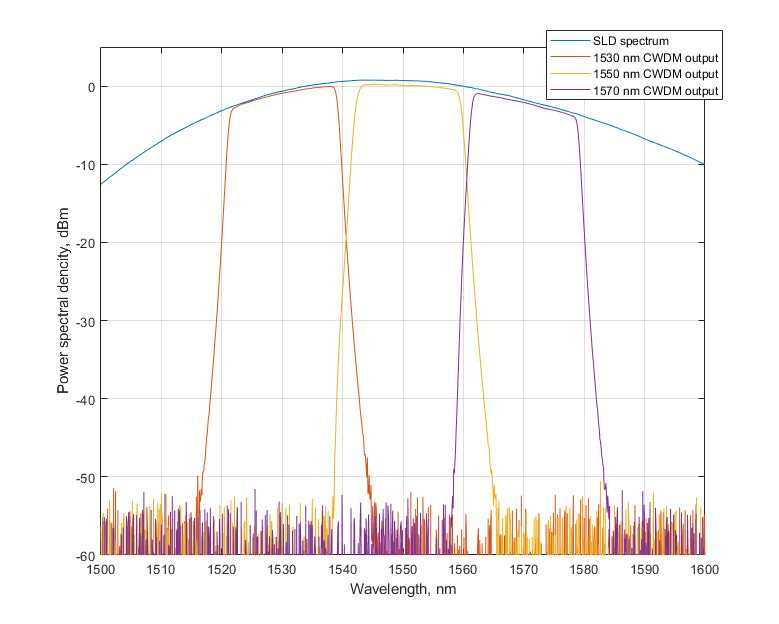
SLD transmission spectra of CWDM modules.

Laser phase noise did not affect the interference significantly in SI compared to other types of distributed fiber sensors [10]. A narrow-bandwidth light source was not needed. In our scheme, we used 15 mW and 9 mW Fabry-Perot laser diodes with central wavelengths at 1550 nm and 1570 nm, respectively, for a 51,800 m loop. The spectra are shown in Figure 6. The CWDM modules had a channel spectral bandwidth of 20 nm, and the SLD transmission spectrum is shown in Figure 7. We did not achieve a perfect match between the laser emission and the CWDM transmission spectra but the power received was enough to achieve a signal-to-noise ratio (SNR) of up to 50 and to detect impacts.

**Figure 8:**
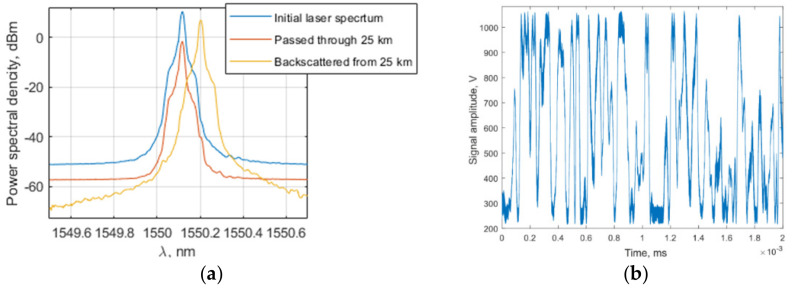
(**a**) Spectra of narrow-band 2 kHz laser radiation, radiation passed through 25 km of fiber and Brillouin scattering, (**b**) Plot of a signal with high Brillouin scattering.

The usage of DWDM modules may narrow down the required spectral range. However, experiments using a narrow-band laser and DWDM modules show a quick nonlinear growth of Brillouin scattering. Figure 8a demonstrates the spectra of narrow-band 2 kHz laser radiation, the radiation passed through 25 km of fiber, and the Brillouin scattering gathered in experiments. This effect leads to signal fluctuations, which made position detection impossible, as shown in Figure 8b.

**Figure 9:**
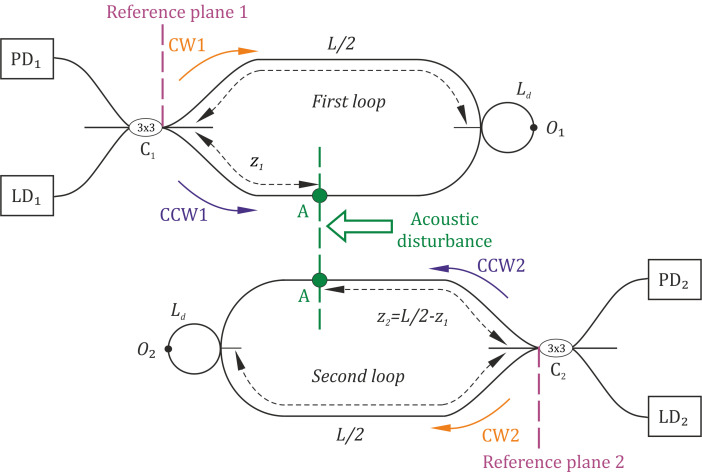
Two-loop diagram for sensitivity analysis.

For a better understanding, the scheme shown in Figure 4 can also be presented as two separate loops, as shown in Figure 9. Light spread with wavelengths *λ*_1_ and *λ*_2_ in the first loop and the second loop, respectively. Light beams in CW and CCW directions interfered at the splitters. We considered C_1_ to be in the first reference plane, and C_2_ to be in the second reference plane. The central points O_1_ and O_2_ were located in half-length of loops.

As two counter Sagnac interferometers were used, a disturbance influenced both loops simultaneously, producing signal phase deviation, which led to interference changes. A disturbance influenced the first loop at a point located at a distance of *z_1_* from the first reference plane. At the same time, it influenced the second loop at a point located at a distance of *z*_2_ from the second reference plane. These two distances are related to total loop length *L* as follows:

(1) $$z_{1}+z_{2}=L/2.$$

External disturbance added a signal phase deviation *φ*(*t*), and the resulting phase difference at the photodetectors from the first and the second loop can be determined as follows:

(2) $$∆\varphi_{1}\left(t,\tau_{CW1},\tau_{CCW1}\right)=\varphi_{CW}\left(t-\tau_{CW1}\right)-\varphi_{\mathrm{CCW}}\left(t-\tau_{CCW1}\right)+\frac{2\pi }{3},$$

(3) $$∆\varphi_{2}\left(t,\tau_{CW2},\tau_{CCW2}\right)=\varphi_{CW}\left(t-\tau_{CW2}\right)-\varphi_{\mathrm{CCW}}\left(t-\tau_{CCW2}\right)+\frac{2\pi }{3},$$

where *φ*_CW_, *φ*_CCW_ is the phase deviation of light propagated in CW and CCW directions; *τ_CW_*_1_, *τ_CCW_*_1_ shows the time during which the radiation travelled in the first loop in CW and CCW directions, respectively, from the disturbance location point to the first reference plane; and *τ_CW_*_2_, *τ_CCW_*_2_ shows the time during which the radiation travelled in the second loop from the disturbance location point to the second reference plane in two directions.

As one can see, at the points of half-loop length, O_1_ and O_2,_ the phase deviations in CW and CCW directions were equal $\left(\varphi_{\mathrm{CW}}\left(t-\tau_{\mathrm{CW}1}\right)=\varphi_{\mathrm{CCW}}\left(t-\tau_{\mathrm{CCW}1}\right);\;\varphi_{\mathrm{CW}}\left(t-\tau_{CW2}\right)=\varphi_{\mathrm{CCW}}\left(t-\tau_{\mathrm{CCW}2}\right)\right)$. Therefore, there was zero phase deviation in the output of the loops $∆\varphi_{1}\left(t,\tau_{CW1},\tau_{CCW1}\right)==∆\varphi_{2}\left(t,\tau_{CW2},\tau_{CCW2}\right)$. It means that a low sensitivity region occurred closer to the center of the loop, the so-called “dead zone”. By including an additional length *L*_d_ in the loops, we moved the points O_1_ and O_2_ outside the sensory region.

The indicated delays are important for signal forming process and can be determined from the lengths in Figure 1:

(4) $$\tau_{CW1}=\frac{\left(L+L_{d}-z_{1}\right)n}{c};\;\tau_{CCW1}=\frac{z_{1}n}{c},\;$$

(5) $$\tau_{CW2}=\frac{z_{2}n}{c};\;\tau_{CCW2}=\frac{\left(L+L_{d}-z_{2}\right)n}{c}$$

The light with gathered phase deviation from point A came to the first reference plane after time delays *τ_CW_*_1_ and *τ_CCW_*_1_, and in both these moments optical power variations occurred at the photodetector, which were the interference signal changes. For the second loop, the interference signal changes occurred in the moments after the *τ_CW_*_2_ and *τ_CCW_*_2_ time delay, when the light came from point A to the second reference plane. Due to a sensor configuration, it is obvious that for any coordinate of point A, the condition (*L*/2 + *L*_d_) > *L*/2 ≥ z_i_, *i* = 1,2 is satisfied. Therefore, the inequalities *τ_CW_*_1_ > *τ_CCW_*_1_ and *τ_CW_*_2_ < *τ_CCW_*_2_ are always correct. Consequently, the interference signal reached the photodetectors PD1 and PD2 at times *τ*_CCW,1_ and *τ*_CW,2_, which were the earliest moments of interference signal changes in both loops. The shift between them is as follows:

(6) $$∆\tau =\frac{z_{2}n}{c}-\frac{z_{1}n}{c}=\frac{\left(z_{2}-z_{1}\right)n}{c}.$$

This value allowed us to calculate a coordinate of point A as its position towards the first reference plane:

(7) $$z_{1}=\frac{∆\tau c}{2n}+\frac{L}{4}$$

Expression (7) is the final equation for determining the disturbance coordinate. As one can see, for disturbance localization it is necessary to gather two interference signals at the outputs of the loop, find the time delay value Δ*τ* between them, and then calculate the distance from the disturbance point to the first reference plane *z*_1_.

## 3. Simulation of a Sensor Sensitivity Distribution

To study sensitivity distribution over the loop of SI, phase change occurring due to acoustic disturbance can be expressed as follows:

(8) $$\varphi \left(t\right)=sin\left(2\pi f_{t}t\right)\cdot A\left(t\right),$$

where $A\left(t\right)$ is the disturbance envelope, we used $A\left(t\right)=a_{0}exp\left(-a_{ac}t\right)\cdot rect\left(\frac{t-\left(t_{0}+\tau_{impact}/2\right)}{\tau_{impact}}\right),$ $a_{0}$ is a constant that determined the magnitude of the disturbance, $a_{ac}$ is an acoustic signal attenuation, $t_{0}$ is the time of impact beginning, $\tau_{impact}$ is the impact duration, and $f_{t}$ is the disturbance frequency.

The phase difference in the first loop output is:

(9) $$∆\varphi_{1}\left(t,∆\tau_{1}\right)=\sin\left(2\pi f_{t}t\right)\cdot A\left(t\right)-\sin\left(2\pi f_{t}\left[t-∆\tau_{1}\right]\right)\cdot A\left(t-∆\tau_{1}\right)\approx \;\approx A\left(t\right)\cdot \left[\sin\left(2\pi f_{t}t\right)-\sin\left(2\pi f_{t}\left[t-∆\tau_{1}\right]\right)\right]=\;=A\left(t\right)\cdot \sin\left(\pi f_{t}∆\tau_{1}\right)\cdot \cos\left(2\pi f_{t}\left[t-\frac{∆\tau_{1}}{2}\right]\right),$$

where a time delay can be calculated as follows:

(10) $$∆\tau_{1}=\tau_{\mathrm{CCW}1}-\tau_{\mathrm{CW}1}=\frac{(L+L_{d}-z_{1})n}{c}-\frac{z_{1}n}{c}=\frac{\left(\left(L+L_{d}\right)-2z_{1}\right)n}{c}$$

For the second loop, the same phase difference can be written as:

(11) $$∆\varphi_{2}\left(t,∆\tau_{2}\right)=\sin\left(2\pi f_{t}t\right)\cdot A\left(t\right)-\sin\left(2\pi f_{t}\left[t-∆\tau_{2}\right]\right)\cdot A\left(t-∆\tau_{2}\right)≅\;≅A\left(t\right)\cdot \left[\sin\left(2\pi f_{t}t\right)-\sin\left(2\pi f_{t}\left[t-∆\tau_{2}\right]\right)\right]=\;=A\left(t\right)\cdot \sin\left(\pi f_{t}∆\tau_{2}\right)\cdot \cos\left(2\pi f_{t}\left[t-\frac{∆\tau_{2}}{2}\right]\right),$$

where a time delay can be calculated as follows:

(12) $$∆\tau_{2}=\tau_{\mathrm{CCW}2}-\tau_{\mathrm{CW}2}=\frac{z_{2}n}{c}-\frac{(L+L_{d}-z_{2})n}{c}=\frac{\left(2z_{2}-\left(L+L_{d}\right)\right)n}{c}$$

Three parts can be highlighted in these expressions. A(t) is the envelope of the external impact and determines the main form of the interference pattern. It has slow changes in time, so $A\left(t\right)\approx A\left(t-∆\tau_{1,2}\right)$. The $\cos\left(2\pi f_{t}\left[t-\frac{∆\tau_{1,2}}{2}\right]\right)$ component has the highest frequency and is responsible for the oscillations of the interference pattern with the frequency of the external impact $f_{t}$. When registering a signal, the intensity of this component will pass through the entire range from minimum to maximum, from −1 to 1. An additional amplitude coefficient for the phase difference and, consequently, the amplitude of the interference pattern at the receiver, is the $\sin\left(\pi f_{t}∆\tau_{1,2}\right)$ component. It becomes zero when the argument is:

$$\pi f_{t}∆\tau_{i}=\pi N,\;where\;N\in Z,\;i=1,2,\;f_{t}∆\tau_{i}=N,\;where\;N\in Z,\;i=1,2,$$

and becomes the maximum when:

$$f_{t}∆\tau_{i}=\frac{1}{2}+N,\;where\;N\in Z,\;i=1,2$$

Thus, the sensitivity graph is a two-dimensional oscillation field, and the maxima, and the minima on the axes of the impact coordinates $z_{1,2}$ and frequency $f_{t}$ will change according to a hyperbolic dependence:

(13) $$f_{t}=\frac{cN}{n\left(\left(L+L_{d}\right)-2z_{1}\right)}\;and\;f_{t}=\frac{cN}{n\left(2z_{2}-\left(L+L_{d}\right)\right)}\;N\in Z\;for\;minima$$

(14) $$f_{t}=\frac{c\left(N+1/2\right)}{n\left(\left(L+L_{d}\right)-2z_{1}\right)}\;and\;f_{t}=\frac{c\left(N+1/2\right)}{n\left(2z_{2}-\left(L+L_{d}\right)\right)}\;N\in Z\;for\;maxima$$

Taking into account the fact that signals usually have a wide spectrum, low impact frequencies and sensor areas close to the middle of the loops are critical for sensing applications

**Figure 10:**
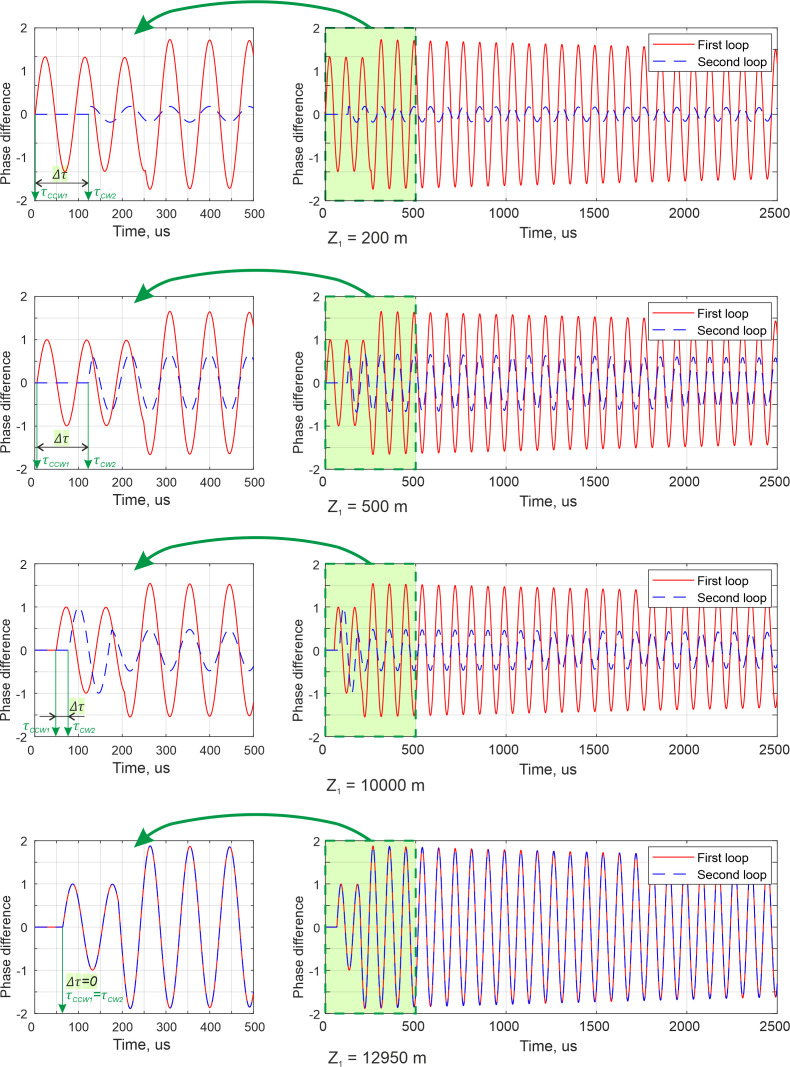
Simulated signal plots for two loops.

Due to the principles of SI signal formation, a phase difference at the sensor loops output differs if the same disturbance influences the loops at points with different coordinates *z_1_*. We considered the sensitivity of the sensor as a maximum phase difference amplitude at the output of the loop, in the presence of an acoustic disturbance. In order to investigate sensitivity distribution through loops and define zones of low sensitivity, where disturbance localization is difficult, we simulated phase difference at the loop outputs ∆*φ*_1_(*t*,∆*τ*_1_), ∆*φ*_2_(*t*,∆*τ*_2_), in a case when a disturbance influences the loops at different distances *z_1_* from the first reference plane. Figure 10 presents diagrams of the phase difference at the output of the first and the second loops, with a length of L = 51,800 m, that arises when a disturbance influences the loops at points distanced for *z*_1_ = 200 m, 500 m, 10,000 m, and 12,950 m from the first reference plane. We assumed that a disturbance had a frequency of *f*_t_ = 11 kHz and produced a phase shift with an amplitude of 2 radians. The ADC sampling frequency was *v_D_* = 25 MHz, the observation time was 2.5 ms, and the loops included additional coils with a length of *L*_d_ = 1000 km in its half-length, in order to move “dead zones” outside the sensory region. The first diagram for *z*_1_ = 200 m shows that this disturbance point is close to the “dead zone” of the first loop. A phase difference amplitude *φ_A_*_1_ = *max*{∆*φ*_1_(*t*,∆*τ*_1_)}-*min*{∆*φ*_1_(*t*,∆*τ*_1_)} was too small in such a case, so the interferential signal amplitude was also small, and the moment when the signal started to change due to a disturbance cannot be distinguished. On the contrary, the phase difference in the second loop was large because the disturbance point was close to the second reference plane, but we needed a start time in both loops for disturbance localization. The point located at a distance of *z*_1_ = 500 m is on the edge of the “dead zone”, as the second diagram shows. The amplitude of the phase difference and an interferential signal amplitude increased, which made it possible to determine the moment when the signal started to change due to a disturbance both in the second loop and in the first loop. The third diagram, showing the point with coordinate *z*_1_ = 10,000 m, shows that phase differences both in the first loop and in the second one are sufficient to localize the disturbance. The fourth diagram shows that for the point with coordinate *z*_1_= *L*/4 = 12,950 m, phase differences at the output of the first and second loops become the same and both are sufficient for disturbance localization. Moreover, in such a case, zero time delay exists (∆*τ* = 0), which is consistent with (7). With a further increase in *z*_1_, when a disturbance point shifts closer to the first loop half-length, the phase difference amplitude increased for the first loop and decreased for the second loop, until the “dead zone” of the second loop was reached, closer to its half-length.

**Figure 11:**
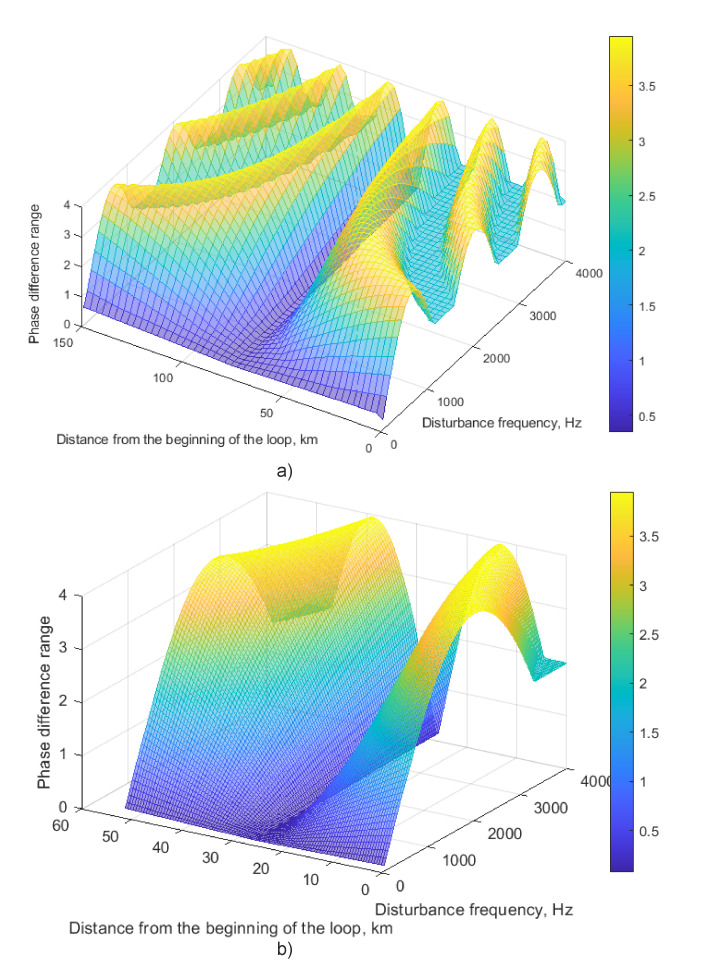
Phase different range depending on disturbance frequency and position: (**a**) L = 150 km, (**b**) L = 51.8 km.

The phase difference range *φ_A_*_1_ in the first loop output, depending on a disturbance position *z*_1_ along the first loop, and its frequency *f_t_* were studied. For this purpose, several disturbances were simulated. Each of them had a fixed frequency and a position along a sensing loop. The time duration was similar to the plots in Figure 10. A simulation of the sensitivity distribution along the first SI loop was carried out depending on two parameters: the coordinate of the disturbance relative to the first reference plane *z*_1_, and the disturbance frequency *f_t_*. For the loop length *L* = 150 km, 50 values of the disturbance coordinate were selected in a range of 0 km to 150 km with a uniform step, as well as 50 values of the disturbance frequency in a range of 40 Hz to 4 kHz with a uniform step. The results are presented in Figure 11a,b.

The sensitivity has a periodical distribution, with a shorter period at high disturbance frequencies, which is consistent with (9) and (11). A “dead zone” is located in the center of the loop, at a distance of *d*_1_ = *L*/2 = 75 km. If a loop has a shorter length, a plot is limited by the length values. When applied to the loop with a length of 51,800 m, the plot from Figure 11a includes only its central region, as shown in Figure 11b. To avoid a low sensitivity region, we used an additional *L_d_* = 1000 m fiber spool that moved the “dead zone” outside of the sensory region. Thus, the ±500 m “dead zone” close to the half-length of the loop was inside the additional coil and throughout the rest of the loop, along the entire sensory region, the disturbance was localized easily. We carried out experimental studies at the coordinate *ž* = 24,500 m with a total loop length of (*L* + *L_d_) =* 51,800 m.

However, the disturbance localization error depends not just on impact spectra, which are determined by the signal shape. It is necessary to take into account the SNR and the ADC sampling rate. To study a mutual dependence of disturbance localization error on these three parameters, an experimental setup was assembled. A series of experiments, with a certain impact shape, were conducted to obtain a coordinate of the disturbance determined by the sensor system.

## 4. Experiment

**Figure 12:**
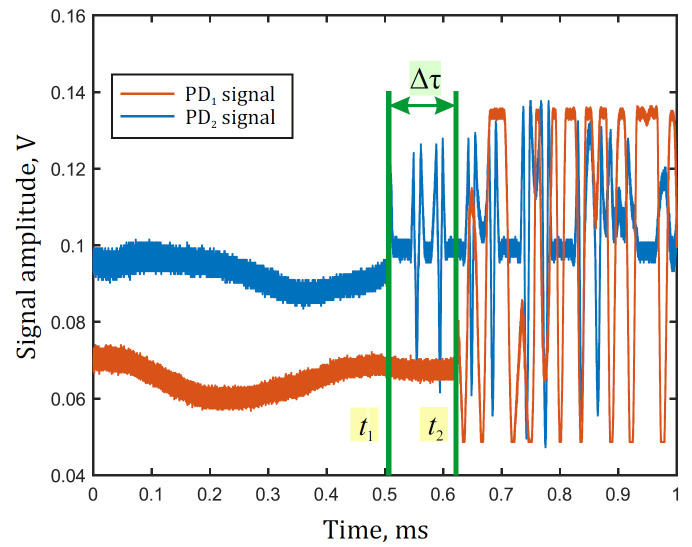
Measured signal plots for two loops.

An experimental setup of the sensor model shown in Figure 4 was assembled to implement the method and analyze disturbance localization error. A piezoelectric transducer (PZT) wound with 20 m of fiber was used to emulate a disturbance. It was positioned at the coordinate *z*_0_ = 25,450 m from the first reference plane, which was measured using a reflectometer. In further experiments, we defined z0 as a true value of the disturbance coordinate. The sensor measured the value of *z*_1_, so we compared *z*_1_ with *z*_0_ to calculate an error of disturbance localization. The total length was *L* = 51,800 m, considering both the coils’ length *L*_c_ = 25,400 m and the delay fiber length *L*_d_ = 1000 m. Pulses from a signal generator with a width of 10 μs produced a deformation with 4 μm amplitude for 20 m of wounded fiber, which were supplied to the PZT. Two FEMTO HCA-S-200M-IN were used as photodetectors. A LeCroy WaveRunner 620Zi oscilloscope operated in the system as an ADC and delivered acquired data to a computer for processing in MATLAB, which was carried out according to the specified method. To investigate the accuracy with which the system identifies localized disturbances, 100 data realizations were acquired and processed. An example of a single data realization obtained from the sensor with *v*_D_ = 25 MHz sampling frequency is shown in Figure 12.

For disturbance localization, we used an adaptive threshold method, consisting of determining the moments of the interference signal modulation beginning with the outputs of loops $t_{1}$ and $t_{2}$. The moments were determined using a signal that exceeded a threshold level, and the time difference, defined as Δ*τ = t*_1_ *− t*_2_, is used in (7).

**Figure 13:**
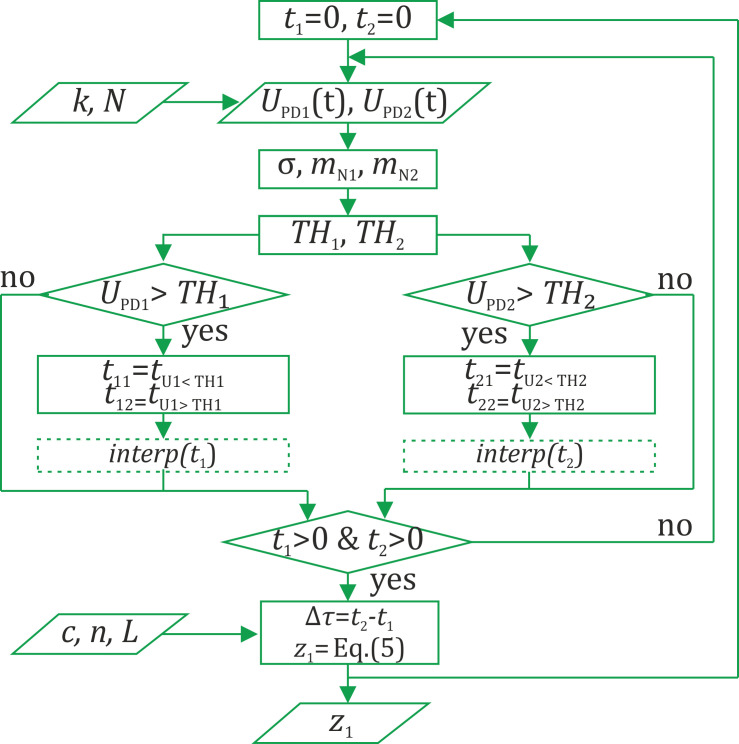
Data processing algorithm.

In the data processing algorithm diagram shown in Figure 13, *U*_PD1,2_ is the level of the signal gathered from PD_1,2_, *TH* is the threshold level, *t*_1,2_ is the beginning moment of interference signal modulation for the first and the second loops, and *z*_1_ is the determined disturbance position.

**Figure 14:**
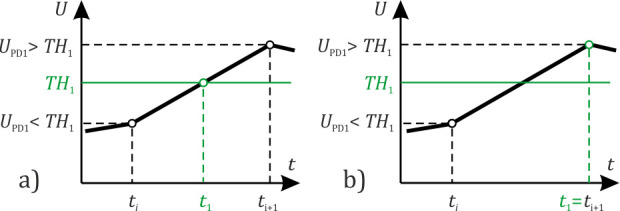
Determination of the detected moments at which the signal exceeds the threshold (**a**) with and (**b**) without interpolation.

TH level is defined as follows:

(15) $$TH_{1,2}\left(v_{D}\right)=m_{N1,2}\left(v_{D}\right)+k\left(v_{D}\right)\cdot \sigma ,$$

where *σ* is the standard deviation, calculated from 100 sample points, and *m*_N1,2_*(ν*_D_*)* is the expected value calculated from N sample points for both data realizations. The number of points *N(ν*_D_*)* depends on sampling frequency *ν*_D_, as well as *k(ν*_D_*)*, which refers to a proportional coefficient. Adjusting the adaptive threshold level allowed us to consider noise level changes and interference signal range changes due to photodetector noise, environmental influence, and polarization instability [8]. Thus, a timely response to disturbances was insured. The moments *t*_1,2_ were determined by linear interpolation between two successive signal points: one occurred before the threshold exceeding, and the other after it, as shown in Figure 14a.

When using an algorithm for defining a time delay ∆*τ*, by determining when the moments of the interference signal modulation begin as specified above, a method for defining these moments is essential. If a time delay ∆*τ* is defined improperly, it causes errors of disturbance localization. A theoretical minimum error of sensor disturbance localization is theoretical spatial resolution *δz*, which can be determined using the sampling rate of the formula *δz = c*/(*n ν*_D_). In a case when a realization is obtained using low sampling frequency, disturbance can be localized with error, limited by SNR in a realization analyzed by the sensor and by a signal shape. Therefore, in practice, an error of disturbance localization is greater than the theoretical spatial resolution due to system noise, and it depends on both SNR and sampling frequency. Implementing interpolation makes it possible to improve the accuracy of determining the interference signal modulation beginning moments when the SNR is high enough, but in a case of a high noise level, this is not enough to overcome the limitations of the algorithm, and interpolation does not provide high-precision results. The SNR value depends on both system state and experimental conditions. The SNR particularly depends on the sampling frequency of ADC. We investigated the SNR dependence on sampling frequency and a disturbance localization error of our system, using the algorithm with and without interpolation for different SNR, i.e., for different sampling frequency values. It allowed us to discover if interpolation is excessive for realizations with high noise and it is appropriate to save computing resources by excluding this stage.

Experiments were carried out with ADC sampling frequencies ranging from 1 to 25 MHz. For each sampling frequency, 100 data realizations with a duration of 1 ms were acquired. First of all, we defined an SNR in a realization as follows:

(16) $$SNR=\frac{S}{\sigma_{N}},$$

where *σ_N_* is noise standard deviation, and *S* is signal amplitude.

**Figure 15:**
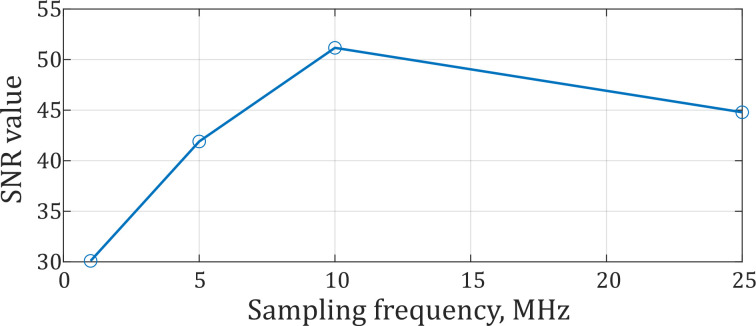
The SNR averaged over 100 realizations for different sampling frequencies.

Signal amplitudes were calculated as a difference between the maximum and the minimum of optical power in the photodetector values when it is modulated due to an acoustic disturbance. Noise standard deviation values were calculated by 162, 829, 1662, and 4162 points for 1, 5, 10, and 25 MHz respectively, which refers to a duration of (1/6) μs. For each certain value of sampling frequency, the SNR was calculated for each realization, and then averaged over 100 realizations. The result is shown in Figure 15. As one can see, the SNR reached the maximum value at *v_D_* = 10 MHz. This value will affect spatial resolution as described in the following.

**Figure 16:**
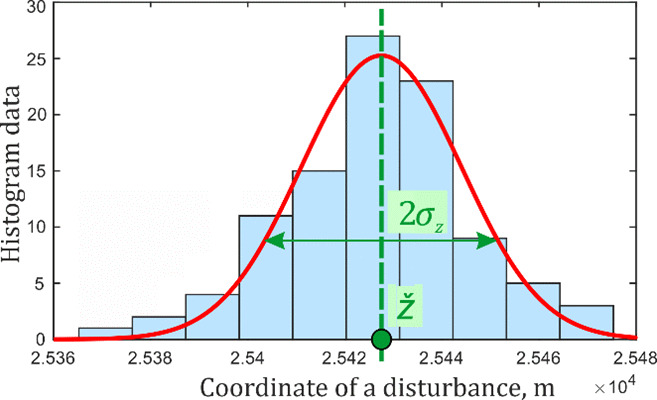
Coordinate determination histogram at ν_D_ = 25 MHz.

For each sampling frequency, 100 data realizations were processed according to the specified algorithm with interpolation. For every sampling frequency value, coordinate distribution histograms were plotted. An example of a histogram for the 25 MHz sampling frequency with its Gaussian approximation is shown in Figure 16.

**Figure 17:**
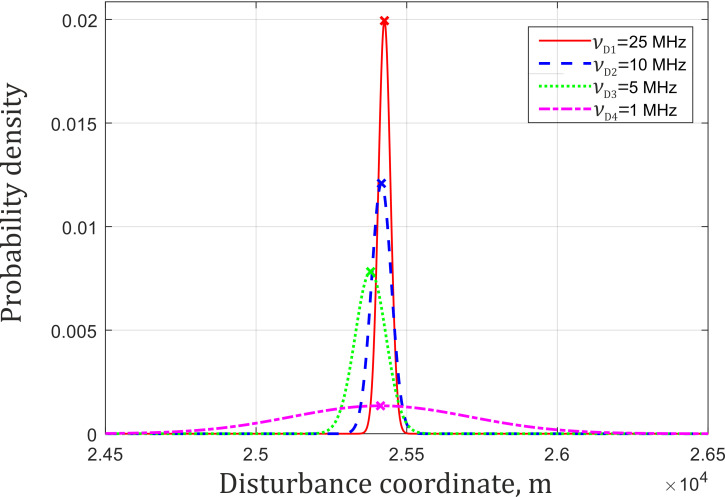
Gaussian approximation of *z*_1_ obtained using interpolation algorithm for different ν_D_ values.

The histogram envelopes of the determined coordinates for the different sampling rates are shown in Figure 17. Table 1 shows values calculated from the results of the experiments performed. Standard deviation *σ*_z_ represents the coordinate determination error for 100 measurements. The expected value *ž* is the average coordinate value for all realizations, ∆*z* is its deviation from the true value *z*_0_.

**Figure 18:**
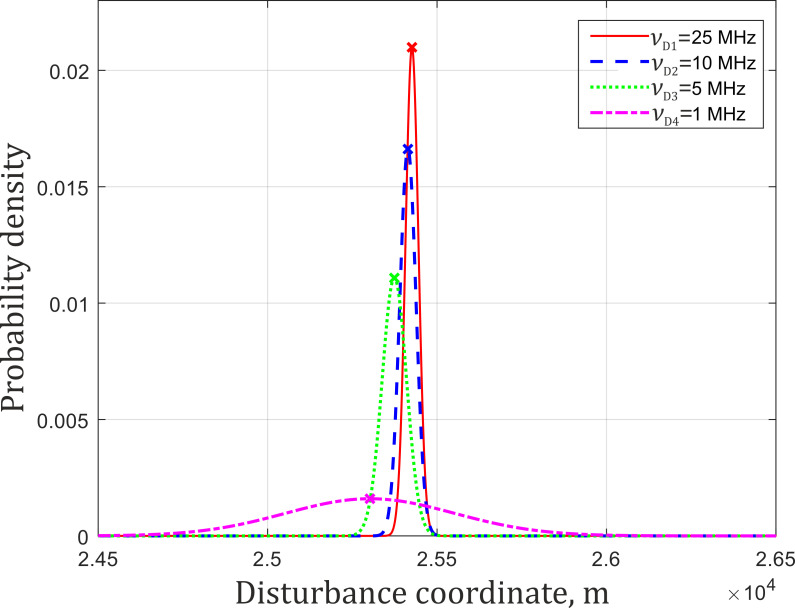
Gaussian approximation of *z*_1_ obtained without interpolation for different ν_D_.

The results indicate that when the sampling frequency grows, the coordinate determination error decreases. However, it increases the theoretical value of *δz*. At *ν*_D_ = 25 MHz, it exceeds the theoretical limit by 2.5 times. Under such conditions, interpolation for coordinate determination is inappropriate. This was verified on the same data using the algorithm from which the interpolation stage was excluded. The obtained results are presented in a similar form in Figure 18 and Table 2.

**Figure 19:**
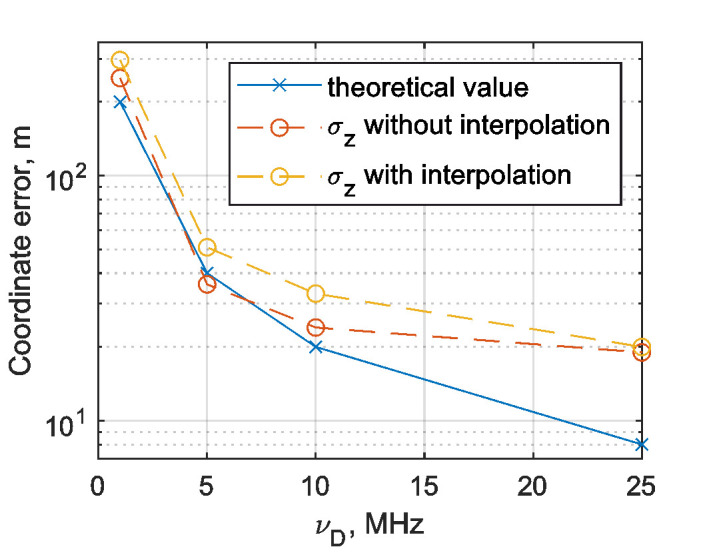
Dependence of disturbance localization error on sampling frequency.

The results show that at low sampling rates, ∆*z* and *σ*_z_ increase, but at high sampling rates, they become similar to the equivalent values obtained when using interpolation. Overall assessment can be carried out based on the graphical presentation of the tables in Figure 19.

## 5. Discussion

Usually, the coordinate determination error *σ*_z_ is comparable to the theoretical expectation for sampling frequencies of up to 10 MHz. Figure 15 and Figure 19 presented that the SNR, for a fixed time duration, has a correlation with the critical value of the coordinate determination error *σ*_z_. The SNR stopped growing at *ν*_D_ = 10 MHz, and at the same sampling frequency *σ*_z_ started to exceed its theoretical limit significantly, which demonstrates that the SNR value influences the disturbance localization error critically. Nevertheless, SNR depends on *ν*_D_, as we have a number of points for the SNR calculation for different *ν*_D_ values, as shown in Equation (16). At *ν*_D_ = 25 MHz, the error in the coordinate determination is significantly higher than the theoretical value, because of the SNR limitation. However, the coordinate determination error decreases when increasing the sampling frequency, and disturbance localization error Δz for *ν*_D_ = 25 MHz is lower than the result for *ν*_D_ = 10 MHz. It means that SNR is not the only limiting factor. Therefore, to achieve a small enough error of disturbance localization in the proposed scheme, it is advisable to use an ADC with a sampling rate of at least 10 MHz and SNR > 44. In addition, in the case of a low sampling rate, interpolation can reduce the average value of Δ*z*, but it is not stable, as shown in Table 1 and Table 2.

In the proposed version of the scheme and algorithm, data processing does not require signal phase unwrapping, so there is no need to use several photodetectors and ADCs at the output of each loop. A disturbance’s coordinates can be determined in other ways, including using correlation, for which it is necessary to restore the original disturbance signal shape. In this case, it is possible to detect the radiation coming from the C_1_ and C_2_ splitter outputs, which are not used in the scheme in Figure 1. Phase restoration can be performed, for example, according to the algorithm [22].

A comparison with previously described sensor configurations is presented in Table 3.

Results show that the suggested scheme does not require phase unwrapping, filtering, and high-cost components such as special fibers, but it demonstrates good localization accuracy, and is competitive with the best existing schemes.

## 6. Conclusions

In this article, we proposed a new scheme for a distributed fiber optic sensor based on a double Sagnac interferometer, and explored its sensitivity for different disturbance frequencies and coordinates, and measured disturbance localization accuracy. We proposed a simple algorithm for determining the coordinates of the disturbance and have confirmed its high-accuracy operation with several experiments. In the laboratory setup, a 25 km sensor fiber was used. A disturbance with stated parameters was localized with different sampling rates and SNR values. The results show that the SNR value is important for reaching a minimal error of disturbance localization. However, increasing the sampling rate allowed the obtention of better accuracy, even with lower SNR. For example, in our setup, we achieved 34 m accuracy (at *ν*_D_ = 10 MHz and SNR = 52) and 24 m accuracy (at *ν*_D_ = 25 MHz and SNR = 44) which is less than 0.1% of the sensing fiber length. This scheme can be used to implement distributed acoustic fiber monitoring systems.

## Figures and Tables

**Table 1:** System parameters in case of interpolation.

*ν_D_*, MHz	*N*(*ν_D_*)	*k*(*ν_D_*)	*δz*, m	*2σ_z_*, m	*ž*, m	∆*z*, m
1	32	5	200	296	25,413	37
5	64	7	40	51	25,380	33
10	64	11	20	33	25,416	34
25	128	11	8	20	25,426	24

**Table 2:** System parameters without interpolation.

*ν*_D_, MHz	δ_z_, m	2σ_z_, m	ž, m	Δz, m
1	200	250	25,302	148
5	40	36	25,374	76
10	20	24	25,414	36
25	8	19	25,426	24

**Table 3:** Comparison of system parameters for reviewed schemes.

Used method	Coordinate Determination Error	Sensor Length	Comments	Ref.
Combination of Michelson and Sagnac interferometers	160 m for 120 km sensor (0.14%)	120 km		[23]
Combination of Mach-Zehnder and Sagnac interferometers	60 m for 61 km sensor (0.1%)	61 km		[24]
SI based on TDE	10 m for a 50 km sensor (0.02%)	50 km	Requires phase unwrapping scheme	[17]
SI based on “null frequencies”	100 m for 50 km sensor (0.2%)	50 km	Highly sensitive to noise	[4]
SI based on TD between countering loops	15 m for a 5 km sensor (0.9%)	5 km	Losses on couplers or Requires PM-fiber	[18,19,20]
Suggested scheme	24 m for 25.4 km sensor (0.1%)	25.4 km		

## Data Availability

The data presented in this study are available on request from the corresponding author.
